# Roles of Calcium/Calmodulin-Dependent Kinase II in Long-Term Memory Formation in Crickets

**DOI:** 10.1371/journal.pone.0107442

**Published:** 2014-09-12

**Authors:** Makoto Mizunami, Yuko Nemoto, Kanta Terao, Yoshitaka Hamanaka, Yukihisa Matsumoto

**Affiliations:** 1 Faculty of Science, Hokkaido University, Sapporo, Japan; 2 Graduate School of Life Sciences, Tohoku University, Sendai, Japan; 3 Graduate School of Life Science, Hokkaido University, Sapporo, Japan; 4 Faculty of Liberal Arts, Tokyo Medical and Dental University, Ichikawa, Japan; Tohoku University, Japan

## Abstract

Ca^2+^/calmodulin (CaM)-dependent protein kinase II (CaMKII) is a key molecule in many systems of learning and memory in vertebrates, but roles of CaMKII in invertebrates have not been characterized in detail. We have suggested that serial activation of NO/cGMP signaling, cyclic nucleotide-gated channel, Ca^2+^/CaM and cAMP signaling participates in long-term memory (LTM) formation in olfactory conditioning in crickets, and here we show participation of CaMKII in LTM formation and propose its site of action in the biochemical cascades. Crickets subjected to 3-trial conditioning to associate an odor with reward exhibited memory that lasts for a few days, which is characterized as protein synthesis-dependent LTM. In contrast, animals subjected to 1-trial conditioning exhibited memory that lasts for only several hours (mid-term memory, MTM). Injection of a CaMKII inhibitor prior to 3-trial conditioning impaired 1-day memory retention but not 1-hour memory retention, suggesting that CaMKII participates in LTM formation but not in MTM formation. Animals injected with a cGMP analogue, calcium ionophore or cAMP analogue prior to 1-trial conditioning exhibited 1-day retention, and co-injection of a CaMKII inhibitor impaired induction of LTM by the cGMP analogue or that by the calcium ionophore but not that by the cAMP analogue, suggesting that CaMKII is downstream of cGMP production and Ca^2+^ influx and upstream of cAMP production in biochemical cascades for LTM formation. Animals injected with an adenylyl cyclase (AC) activator prior to 1-trial conditioning exhibited 1-day retention. Interestingly, a CaMKII inhibitor impaired LTM induction by the AC activator, although AC is expected to be a downstream target of CaMKII. The results suggest that CaMKII interacts with AC to facilitate cAMP production for LTM formation. We propose that CaMKII serves as a key molecule for interplay between Ca^2+^ signaling and cAMP signaling for LTM formation, a new role of CaMKII in learning and memory.

## Introduction

Ca^2+^/calmodulin (CaM)-dependent protein kinase II (CaMKII) belongs to the family of serine/threonine-specific protein kinases and is regulated by the Ca^2+^/CaM complex [1], [2]. It is an important mediator of many biological processes, including learning and memory [1]. A notable feature of this kinase is that it can act as a protein switch; once activated by Ca^2+^/CaM, the enzyme can be autophosphorylated at T286, which is an event that makes CaMKII activity persist even after the Ca^2+^ concentration falls to a baseline level, an ideal feature for memory retention.

The roles of CaMKII in learning and memory have been well documented in mammals [1], [2]. CaMKII is a major protein in post-synaptic density in the rat hippocampus and is activated by changes in Ca^2+^ concentration during long-term potentiation (LTP) [3]. Pharmacological blockade of CaMKII or knockout of the *CaMKII* gene results in deficits in LTP of synaptic activity in the hippocampus and impairment of hippocampus-dependent spatial learning and memory in mice [4], [5]. If persistent activation of CaMKII is prevented by a point mutation that blocks autophosphorylation of threonine at position 286, LTP induction is prevented and mice show profound memory impairments [6]. These results indicate that prolonged activation of CaMKII is necessary for neural plasticity underlying some forms of learning.

Invertebrates such as insects and mollusks have been used as model animals to study molecular and cellular mechanisms of learning and memory [7]–[11], but knowledge of the roles of CaMKII in invertebrate learning and memory is still limited. In mollusks, CaMKII participates in short-term synaptic potentiation [12], intermediate-term sensitization [13] and consolidation of long-term memory (LTM) [14], but its molecular mechanisms are not well understood. In the courtship conditioning in the fruit-fly *Drosophila*, in which a male fly exposed to a previously mated female exhibits suppression of courtship to a virgin female, inhibition of CaMKII in the central complex and parts of the lateral protocerebrum impairs memory formation [15], [16]. In olfactory conditioning in fruit-flies, it has been reported that synthesis of synaptic proteins including CaMKII in Kenyon cells (intrinsic neurons) of the mushroom body, a multisensory association center participating in olfactory learning [8], [17], is necessary for formation of LTM [18]–[20]. In learning of cockroaches to associate an odor with a visual cue, phosphorylated forms of CaMKII increases in pre- and postsynaptic structures in the calyx of the mushroom body after learning [21]. In olfactory conditioning in honey bees, we recently reported that pharmacological blockade of CaMKII impairs formation of protein synthesis-dependent LTM [22]. Despite of the importance in LTM formation as described above, the location of CaMKII in biochemical cascades underling LTM formation remains unexplored.

In insects, cAMP signaling plays critical roles in the formation of protein synthesis-dependent olfactory long-term memory (LTM) [23]. Activation of adenylyl cyclase (AC) leads to production of cAMP and subsequent activation of protein kinase A (PKA), which phosphorylates the transcription factor cAMP responsive element-binding protein (CREB). The CREB leads to transcription and translation of synaptic proteins necessary to elevate efficacy of synaptic transmission that underlies LTM [23]. The NO/cGMP system also plays critical roles in LTM formation in olfactory learning in crickets [24], [25], honey bees [26], [27] and cockroaches [28] and in visual learning in crickets [29]. In crickets, results of our pharmacological studies suggested that cAMP signaling is a downstream target of NO/cGMP signaling, cyclic nucleotide-gated (CNG) channel and Ca^2+^/CaM signaling [24], [25], which provides a solid basis for further studies on signaling cascades underlying LTM formation.

In this study, we investigated the roles of CaMKII in LTM formation in crickets and examined the relationship of CaMKII with other signaling pathways.

## Materials and Methods

### Insects

Adult male crickets, *Gryllus bimaculatus*, at 1–2 weeks after the imaginal molt were used. They were reared in a 12 h∶12 h light: dark cycle (photophase: 8:00–20:00) at 27±2°C and were fed a diet of insect pellets and water *ad libitum*. Four days before the start of the experiment, a group of 20–30 animals was placed in a container and fed a diet of insect pellets *ad libitum* but deprived of drinking water to enhance their motivation to search for water. On the day of the experiment, they were individually placed in 100-ml glass beakers.

### Conditioning

We used classical conditioning and operant testing procedures described previously [30], [31]. Banana or apple odor was used as conditioned stimulus (CS), and water was used as unconditioned stimulus (US). A syringe containing water was used for conditioning. A filter paper soaked with banana or apple essence was attached to the needle of the syringe. The filter paper was placed above the cricket's head so as to present an odor, and then water reward was presented to the mouth. After the conditioning trials, the air in the beaker was ventilated. The crickets were subjected to one or three pairing trials. For the latter, the inter-trial interval (ITI) was 5 min.

### Preference test

The procedure for the odor preference test was described previously [30], [31]. In short, all groups of animals were subjected to odor preference tests before and after conditioning. The floor of the test chamber of the test apparatus has two holes that connected the chamber with two odor sources. Each odor source consisted of a plastic container containing a filter paper soaked with 3 µl solution of banana or apple essence, covered with fine gauze net. Three containers were mounted on a rotative holder and two of three odor sources could be located simultaneously beneath the holes of the test chamber. Before the odor preference test, a cricket was transferred to the waiting chamber and left for about 4 min to become accustomed to the surroundings. Then the cricket was allowed to enter the test chamber and the test started. Two min later, the relative positions of the banana and apple sources were exchanged by rotating the container holder. The preference test lasted for 4 min. If the total time of visits of an animal to either source was less than 10 sec, we considered that the animal was less motivated to visit odor sources, possibly due to a poor physical condition, and the data were rejected. No significantly different levels of conditioning effects were obverted between groups in which banana odor or apple odor was used as CS, and thus we pooled the data from the two groups.

### Pharmacology

Each animal was injected with 3 µl of cricket saline [32] containing drugs into the hemolymph through a hole made on the head capsule using a microsyringe. 8-Bromoguanosine 3',5'-cyclic monophosphate (8-br-cGMP), 8-bromoadenosine 3',5'-cyclic monophosphate (8-br-cAMP), dimethyl sulphoxide (DMSO), ionomycin, forskolin, 1,9-dideoxyforskolin, KN-62 and KN-93 were purchased from Sigma-Aldrich (Tokyo, Japan). Ionomycin, forskolin, 1,9-dideoxyforskolin, KN-62 and KN-93 were dissolved in saline containing 1% DMSO, and other drugs were dissolved in cricket saline.

### Data analysis

We considered a cricket visited an odor source when the cricket probed the gauze net covering the odor source with its mouth or pulpi. The time spent visiting each odor source was measured cumulatively. Relative preference foe the rewarded odor was determined using the preference index (PI), defined as t_r_/(t_r_+t_nr_)x100 (%), where t_r_ was the time spent exploring the odor paired with reward and t_nr_ was the time spent exploring the odor not used in training. Because many of our data violated the assumption of normal distribution, we used non-parametric tests. We compared odor preferences after training with those before training in each animal group by the Wilcoxon signed-rank test (WCX test). We also compared preferences after training between different groups by the Mann-Whitney U test (M-W test). For multiple comparisons, Holm's method was used for adjusting the P value. We found no significant different odor preferences among the different groups of animals before training (Kruskal-Wallis test, p>0.05).

## Results

### Effect of CaMKII inhibitor on LTM formation

We have shown that crickets subjected to 2 or 3 conditioning trials to associate an odor with water reward (with an inter-trial interval of 5 min) exhibit memory that lasts for at least one day, which is characterized as protein synthesis-dependent LTM [30], [32]. In contrast, crickets subjected to 1-trial conditioning exhibit memory that lasts for several hours, which does not require protein synthesis and is characterized as mid-term memory (MTM).

**Figure 1:**
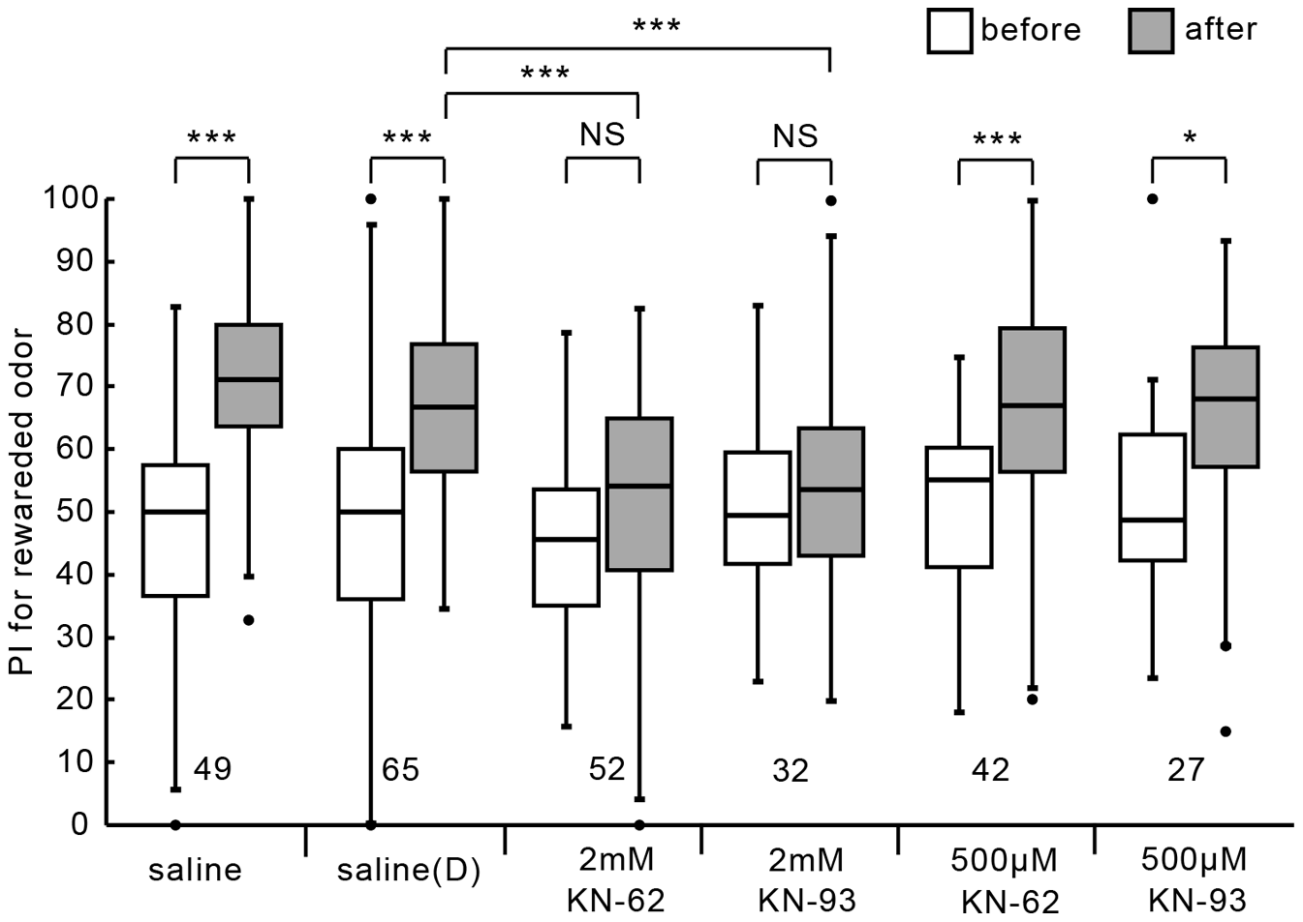
Effects of CaMKII inhibitors on 1-day retention. Two control groups were each injected with 3 µl of saline or saline containing 1% DMSO (designated as saline (D)) 20 min prior to 3-trial conditioning. Another four groups were each injected with 3 µl of saline containing 2 mM KN-62, 2 mM KN-93, 500 µM KN-62 or 500 µM KN-93 (dissolved in 1% DMSO) 20 min prior to 3-trial conditioning. Relative preference between the rewarded odor and control odor was tested before and at 1 day after training. Preferences indexes (PIs) for the rewarded odor before (white bars) and after (grey bars) training are shown as box and whisker diagrams. The line in the box is the median and the box represents the 25–75 percentiles. Whiskers extend to extreme values as long as they are within a range of 1.5× box length from the upper or lower quartiles. Any data not included between the whiskers are plotted as outliers with dots. Odor preferences before and after training are compared by WCX test. Odor preferences after training of different groups were compared by the M-W test. The results of statistical comparisons are shown by asterisks (*** P<0.001, ** P<0.01, * P<0.05, NS P>0.05, adjusted by Holm's method). The number of animals tested is shown at each data point in this figure and in subsequent figures.

We first studied the effects of KN-62 and KN-93, which specifically inhibit insect CaMKII [33], [34], on LTM formation. KN-62 and KN-93 bind to the CaM binding site of the regulatory subunit of CaMKII and prevent its activation by CaM. Four groups of animals were each injected with 3 µl of cricket saline containing 500 µM KN-62, 2 mM KN-62, 500 µM KN-93 or 2 mM KN-93 into the hemolymph of the head at 20 min prior to 3-trial conditioning. The drugs were dissolved in 1% DMSO, and another two control groups were each injected with 3 µl of saline or saline containing 1% DMSO (saline (D)) at 20 min prior to 3-trial conditioning. The relative preference of animals between the conditioned odor and control odor was tested before and 1 day after conditioning. The groups injected with saline or saline (D) exhibited significantly increased preference for the conditioned odor at 1 day after conditioning compared to that before conditioning (Fig. 1; saline group: W = 44, p<0.000001, saline (D) group: W = 231, p<0.000001, WCX test, adjusted by Holm's method, sample numbers shown in legends). On the other hand, the group injected with 2 mM KN-62 or 2 mM KN-93 exhibited no significant level of memory retention at 1 day after conditioning (KN-62: W = 538, p = 0.17, KN-93: W = 229, p = 0.52, WCX test). Between-group comparison also showed that the level of 1-day retention of KN-62 group or KN-93 group was significantly less than that of the saline (D) group (KN-62: U = 2468 p = 0.000040; KN-93: U = 1527, p = 0.00038, M-W test, adjusted by Holm's method). The effect of the CaMKII inhibitors was dose-dependent: The group injected with 500 µM KN-62 or 500 µM KN-93 exhibited a significant level of 1-day retention (KN-62: W = 160, p = 0.00047; KN-93: W = 86, p = 0.012, WCX test). The results indicate that CaMKII participates in formation of 1-day memory. The relationship between drug concentration and dose (mg/Kg) is shown in Table 1.

**Table 1:** Drug doses used in the present study.

Drug	Concentration (mM)	Dose (mg/Kg)
KN-62	2.0	5.1
KN-93	2.0	4.2
8br-cGMP	0.50	0.79
8br-cAMP	0.20	0.44
ionomycin	0.20	0.53
forskolin	0.20	0.29
1,9-dideoxyforskolin	0.20	0.27

Injected volume: 3 µl; averaged body weight: 850 mg.

**Figure 2:**
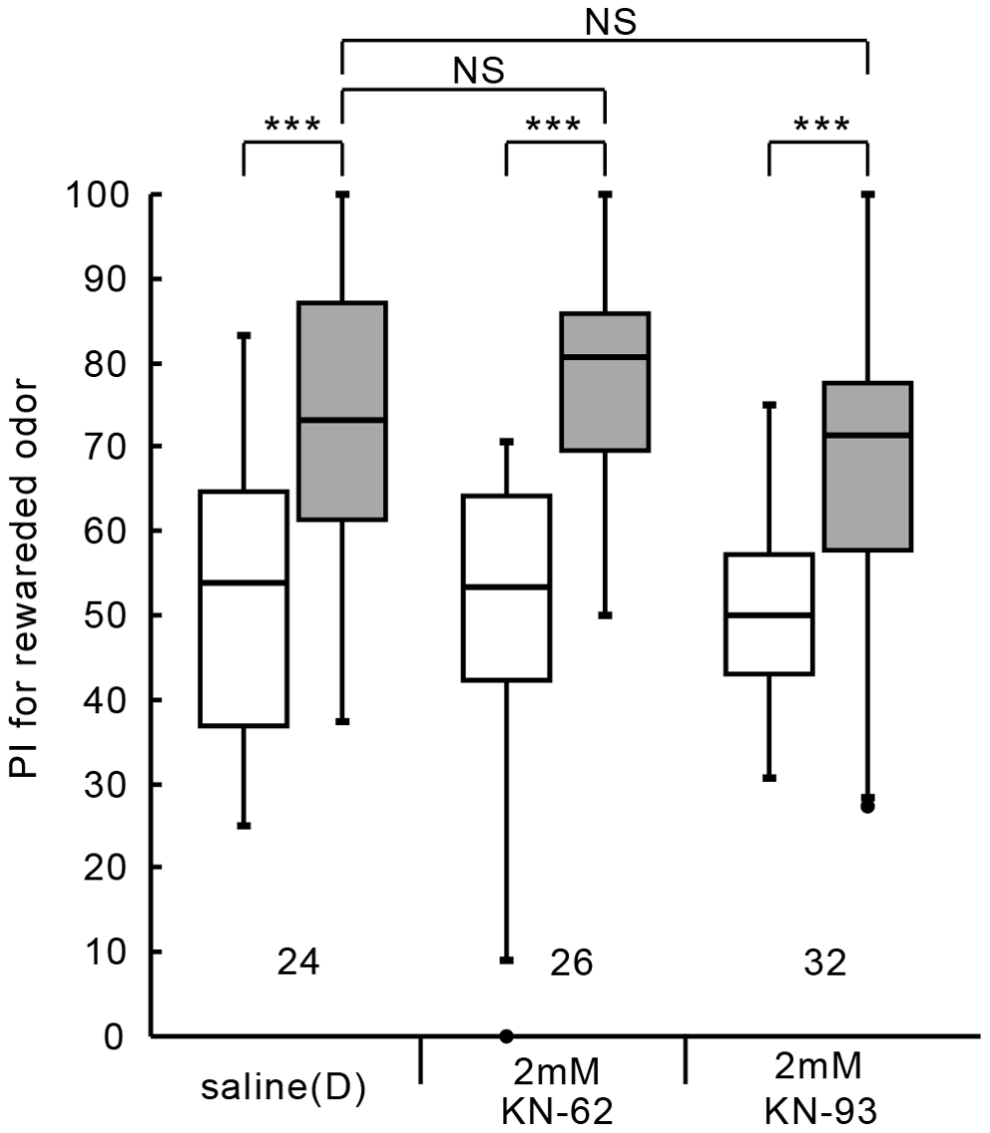
No effects of CaMKII inhibitors on 1-hour retention. Three groups of animals were each injected with 3 µl of saline containing 1% DMSO (designated as saline (D)) or saline containing 2 mM KN-62 or 2 mM KN-93 (dissolved in 1% DMSO) 20 min prior to 3-trial conditioning. Relative preference between the rewarded odor and control odor was tested before and at 1 hour after training. PIs for the rewarded odor before (white bars) and after (grey bars) training are shown as box and whisker diagrams. Odor preferences before and after training are compared by WCX test. Odor preferences after training of different groups were compared by the M-W test. The results of statistical comparisons are shown by asterisks (*** P<0.001, NS P>0.05, adjusted by Holm's method).

We next tested whether KN-62 or KN-93 impairs formation of 1-hour memory (MTM). The group injected with 3 µl of saline containing 2 mM KN-62 or 2 mM KN-93 at 20 min prior to 3-trial conditioning exhibited a significant level of 1-hour memory retention (Fig. 2; KN-62: W = 8, p = 0.0000037, KN-93: W = 48, p = 0.000011, WCX test, adjusted by Holm's method), as did the control group injected with saline (D) (W = 24, p = 0.00027, WCX test, adjusted by Holm's method). Between-group comparison showed that the level of 1-hour retention of KN-62 group or KN-93 group did not significantly differ from that of the control group (KN-62: U = 278, p = 0.51; KN-93: U = 461, p = 0.41, M-W test, adjusted by Holm's method). The results indicate that CaMKII is not required for formation of 1-hour memory. Animals injected with KN-62 or KN-93, or any other drugs used in this study, exhibited normal responses to water US in conditioning trials: They drank water eagerly when water was presented to the mouth, as did intact or saline-injected animals. Drug-injected animals also exhibited normal locomotory activity and exploration of odor sources during testing. The results indicate that KN-62 or KN-93 did not impair 1) sensory and motor functions necessary for performing conditioned response, 2) initial acquisition of memory or 3) memory retention up to 1 hour after conditioning. We conclude that CaMKII specifically participates in LTM formation.

### Effective time window of CaMKII inhibitor

**Figure 3:**
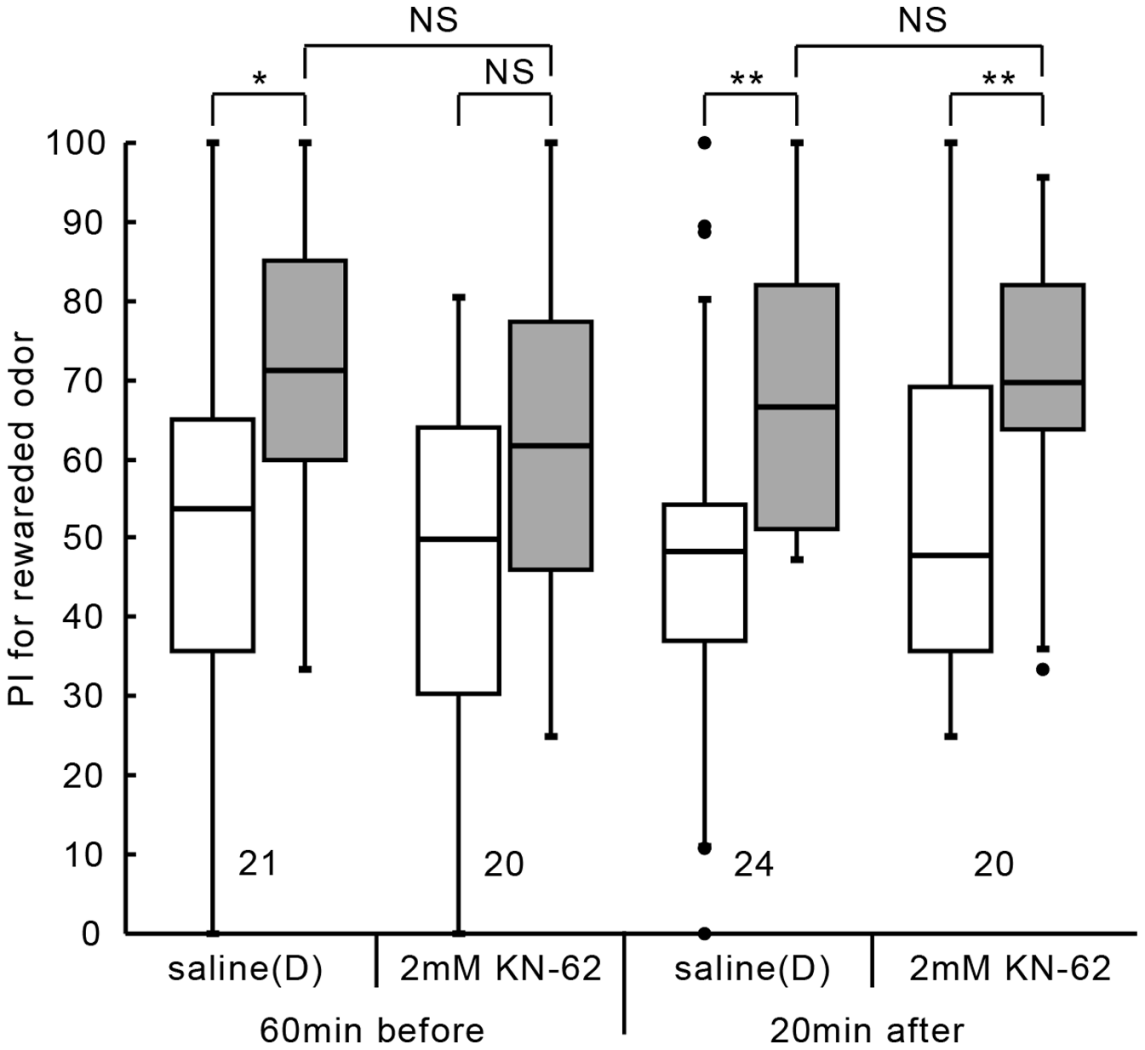
Effective time window of injection of a CaMKII inhibitor. Two groups of animals were each injected with 3 µl of saline containing 1% DMSO (saline (D)) or saline containing 2 mM KN-62 (dissolved in 1% DMSO) 60 min prior to 3-trial conditioning. Another two groups of animals were each injected with 3 µl of saline (D) or saline containing 2 mM KN-62 20 min after 3-trial conditioning. Relative preference between the rewarded odor and control odor was tested before and at 1 day after training. PIs for the rewarded odor before (white bars) and after (grey bars) training are shown as box and whisker diagrams. Odor preferences before and after training were compared by WCX test and odor preferences after training of different groups were compared by the M-W test. The results of statistical comparisons are shown by asterisks (** P<0.01, * P<0.05, NS P>0.05, adjusted by Holm's method).

Next, we investigated the effective time window of the injected CaMKII inhibitor. The group injected with 3 µl of saline containing 2 mM KN-62 at 20 min after conditioning exhibited a significant level of 1 day memory retention (Fig. 3; W = 17, p = 0.0054; WCX test, adjusted by Holm's method), but that at 60 min before conditioning was marginal (W = 54, p = 0.12; WCX test, adjusted by Holm's method). The control group injected with saline (D) at 60 min before conditioning or that 20 min after conditioning exhibited a significant level of memory retention (60 min before: W = 26, p = 0.010; 20 min after: W = 45.5, p = 0.0059, WCX test, adjusted by Holm's method). Between-group comparison showed that the level of one-day retention of the group injected at 60 min prior to conditioning or that injected at 20 min after conditioning did not significantly differ from that of saline-injected group (60 min before: U = 264, p = 0.16; 20 min after: U = 214.5, p = 0.56, M-W test, adjusted by Holm's method). In short, injection of KN-62 at 20 min prior to conditioning, but not at 20 min after conditioning, impaired LTM formation and that at 60 min prior to conditioning had a marginal effect. The results suggest that normal activities of CaMKII are needed at the time of conditioning.

### Relationship between CaMKII and cGMP signaling or cAMP signaling for LTM formation

**Figure 4:**
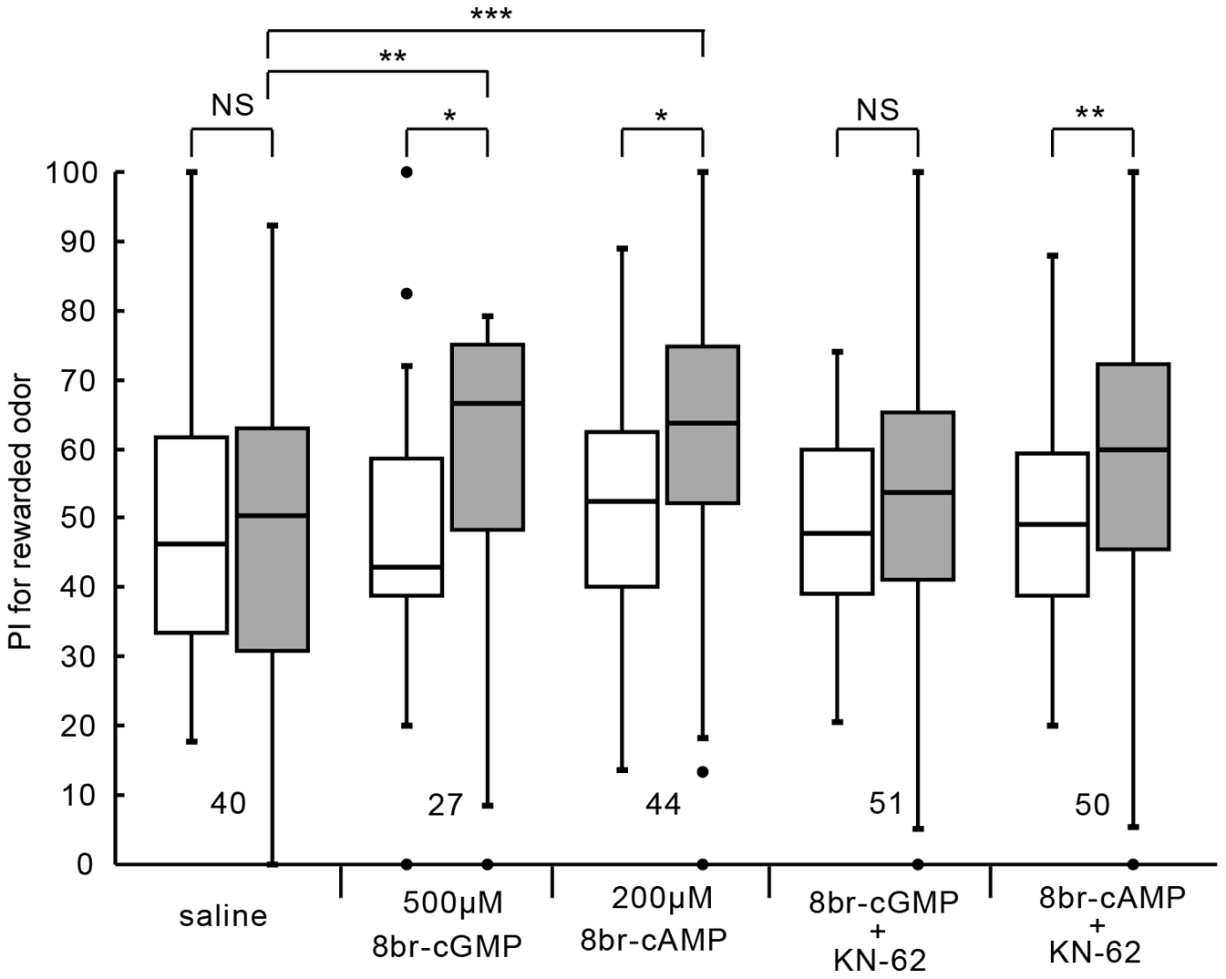
Effects of co-injection of a CaMKII inhibitor and 8br-cGMP or CaMKII inhibitor and 8br-cAMP on LTM formation. Five groups of animals were each injected with 3 µl of saline or saline containing 8br-cGMP (500 µM), 8br-cAMP (200 µM), 8br-cGMP (500 µM) and KN-62 (2 mM) or 8br-cAMP (500 µM) and KN-62 (2 mM) 20 min prior to 1-trial conditioning. KN-62 was dissolved in DMSO (1%). Relative preference between the rewarded odor and control odor was tested before and at 1 day after training. PIs for the rewarded odor before (white bars) and after (grey bars) training are shown as box and whisker diagrams. Odor preferences before and after training are compared by WCX test. Odor preferences after training of different groups were compared by the M-W test. The results of statistical comparisons are shown by asterisks (*** P<0.001, ** P<0.01, * P<0.05, NS P>0.05, adjusted by Holm's method).

We have shown that crickets injected with a membrane-permeable cAMP analog or cGMP analog prior to 1-trial conditioning exhibited 1-day retention when peppermint odor is used as CS [24]. We first confirmed this finding by using apple odor or banana odor as CS. The group injected with saline at 20 min prior to 1-trial conditioning exhibited no significant level of 1-day retention (Fig. 4; W = 380, p = 0.69, WCX test). In contrast, the group injected with 3 µl of saline containing 8br-cGMP (500 µM) or 8br-cAMP (200 µM) at 20 min prior to 1-trial conditioning exhibited a significant level of 1-day retention (8br-cGMP: W = 81, p = 0.016; 8br-cAMP: W = 283, p = 0.027, WCX test, adjusted by Holm's method). Between-group comparison also showed that the preference for the conditioned odor of the 8br-cGMP group or the 8br-cAMP group was significantly greater than that of the saline group (8br-cGMP: U = 294, p = 0.0051; 8br-cAMP: U = 469.5, p = 0.00072, M-W test, adjusted by Holm's method). The effect was dose-dependent: the group injected with 3 µl of saline containing 200 µM 8br-cGMP or 50 µM 8br-cAMP at 20 min prior to 1-trail conditioning exhibited no significant level of 1-day retention (data not shown).

Next, we examined the relationship between CaMKII and other biochemical processes involved in LTM formation. We have evidence that in crickets LTM is formed by serial activation of NO/cGMP signaling, CNG channel, Ca^2+^/CaM, and cAMP signaling [24], [25]. In order to evaluate whether CaMKII is upstream or downstream of cGMP signaling or cAMP signaling, we studied the effect of co-injection of 8br-cGMP and KN-62 or that of 8br-cAMP and KN-62 at 20 min prior to 1-trial conditioning. The group co-injected with 8br-cGMP and KN-62 exhibited no significant level of 1-day retention (W = 608, p = 0.61, WCX test). In contrast, the group co-injected with 8br-cAMP and KN-62 exhibited a significant level of 1-day retention (W = 336, p = 0.0060, WCX test). The findings that KN-62 impaired LTM induction by a cGMP analogue but not that by a cAMP analogue suggest that CaMKII is downstream of cGMP production and upstream of cAMP production in signaling cascades for LTM formation.

### Activation of CaMKII by Ca^2+^ for LTM formation

**Figure 5:**
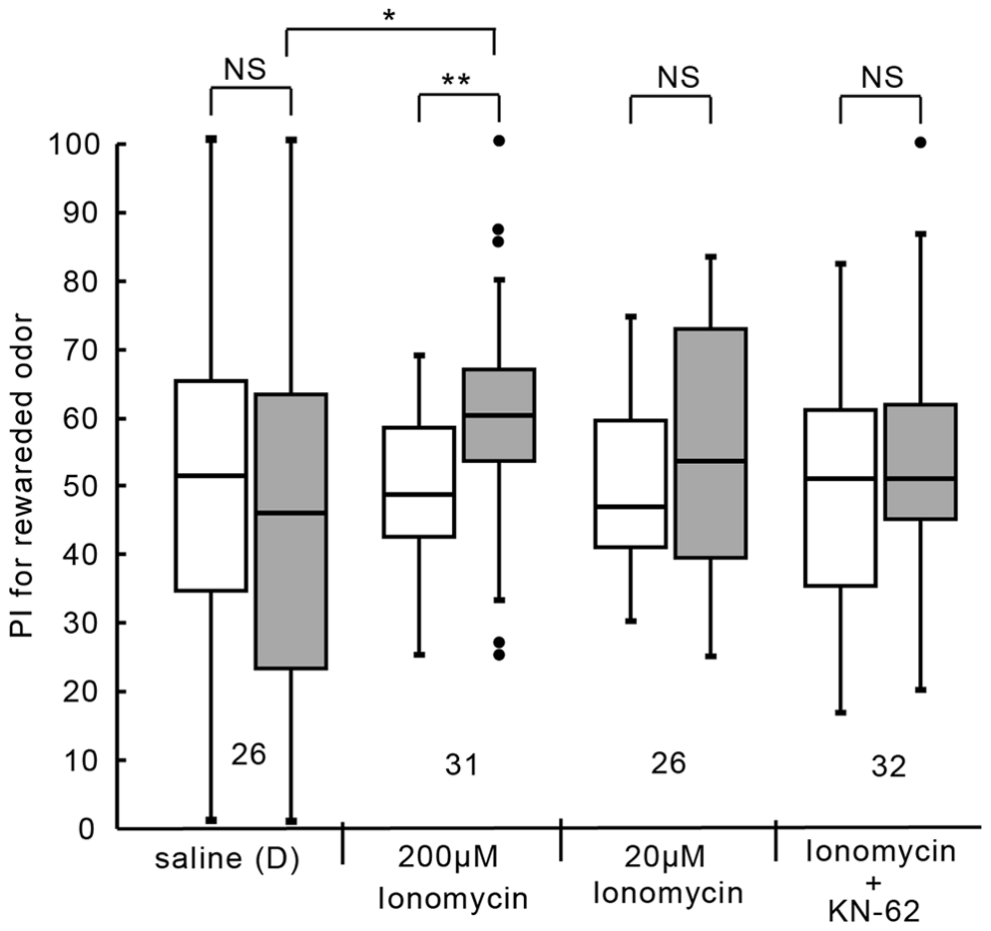
Effects of co-injection of a CaMKII inhibitor and ionomycin on LTM formation. Four groups of animals were each injected with 3 µl of saline or saline containing 20 µM ionomycin, 200 µM ionomycin, or 200 µM ionomycin and 2 mM KN-62 20 min prior to 1-trial conditioning. The saline contained 1% DMSO. Relative preference between the rewarded odor and control odor was tested before and at 1 day after training. PIs for the rewarded odor before (white bars) and after (grey bars) training are shown as box and whisker diagrams. Odor preferences before and after training are compared by WCX test. Odor preferences after training of different groups were compared by the M-W test. The results of statistical comparisons are shown by asterisks (** P<0.01, * P<0.05, NS P>0.05, adjusted by Holm's method).

We have suggested that Ca^2+^ entry and the resulting activation of CaM are upstream of AC activation for LTM formation [24]. We next studied whether CaMKII is stimulated by Ca^2+^ entry for LTM formation. The control group injected with saline at 20 min prior to 1-trial conditioning exhibited no significant level of 1-day memory retention (Fig. 5; W = 218, p = 0.29, WCX test). The group injected with 3 µl of saline containing 200 µM ionomycin, a Ca^2+^ ionophore, at 20 min prior to 1-trial conditioning exhibited a significant level of 1-day retention (W = 93, p = 0.0051, WCX test, adjusted by Holm's method). The level of 1-day retention of the ionomycin group was significantly greater than that of saline group (U = 253.5, p = 0.034, M-W test, adjusted by Holm's method). The effect of ionomycin was dose-dependent: there was no significant level of 1-day retention in the group injected with 3 µl of saline containing 20 µM ionomycin (W = 117, p = 0.14, WCX test). In another group, in which ionomycin and KN-62 were co-injected at 20 min prior to 1-trial conditioning, no significant level of 1-day retention was observed (W = 194, p = 0.19, WCX test). These results indicate that an increase of Ca^2+^ concentration stimulates CaMKII for LTM formation.

### Relationship between CaMKII and AC for LTM formation

**Figure 6:**
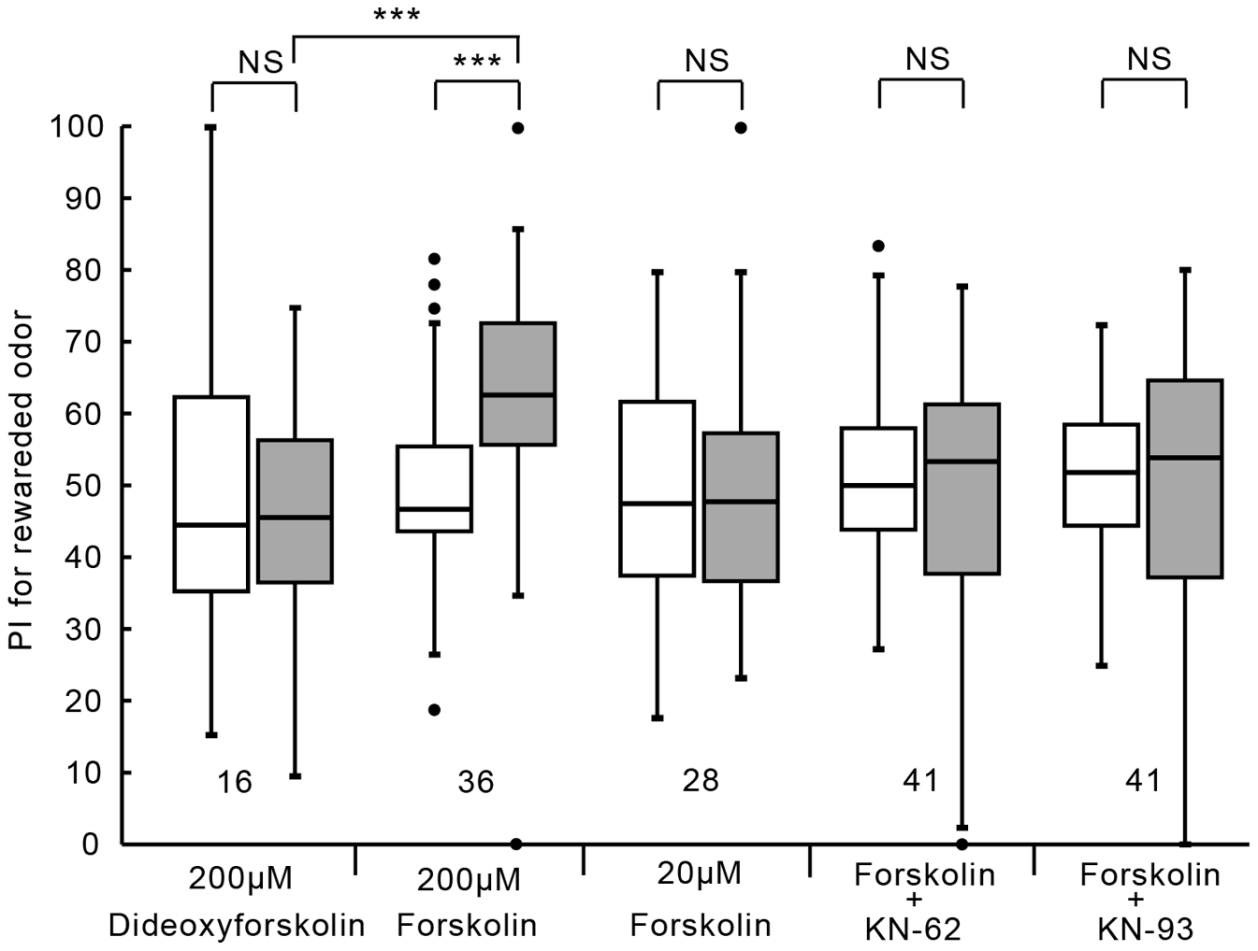
Effects of co-injection of a CaMKII inhibitor and forskolin on LTM formation. Five groups of animals were each injected with 3 µl of saline containing 200 µM 1,9-dideoxyforskolin, 200 µM forskolin, 20 µM forskolin, 200 µM forskolin and 2 mM KN-62 or 200 µM forskolin and 2 mM KN-93 20 min prior to 1-trial conditioning. All of these drugs were dissolved in DMSO (1%). Relative preference between the rewarded odor and control odor was tested before and at 1 day after training. PIs for the rewarded odor before (white bars) and after (grey bars) training are shown as box and whisker diagrams. Odor preferences before and after training are compared by WCX test. Odor preferences after training of different groups were compared by the M-W test. The results of statistical comparisons are shown by asterisks (*** P<0.001, NS P>0.05, adjusted by Holm's method).

Finally, we studied the relationship between CaMKII and AC for LTM formation. We have shown that animals injected with forskolin, an activator of AC, prior to 1-trial conditioning exhibit 1-day retention when peppermint odor is used as CS [24]. We first confirmed this finding with apple odor or banana odor as CS. The group injected with 3 µl of saline containing 200 µM forskolin exhibited a significant level of 1-day retention (Fig. 6; W = 81, p = 0.000070, WCX test, adjusted by Holm's method). A control group injected with 3 µl of saline containing 200 µM 1,9-dideoxyforskolin, an analogue of forskolin that does not activate AC [35], exhibited no significant level of 1-day retention (W = 54, p = 0.50, WCX test). Between-group comparison also showed that the level of 1-day retention of forskolin group was significantly greater than that of 1,9-dideoxyforskolin group (U = 479, p = 0.00032, M-W test, adjusted by Holm's method). The effect of forskolin was dose-dependent: the group injected with 3 µl of saline containing 20 µM forskolin exhibited no significant level of 1-day retention (W = 224, p = 0.65, WCX test). Another two groups were each co-injected with 200 µM forskolin and KN-62 or 200 µM forskolin and KN-93 at 20 min prior to 1-trial conditioning. Both groups exhibited no significant level of 1-day retention (forskolin+KN-62: W = 439.5, p = 0.91; forskolin+KN-93: W = 385, p = 0.74 WCX test). The finding that blockade of CaMKII by KN-62 or KN-93 prevents induction of LTM by folskolin is surprising, because blockade of CaMKII by KN-62 does not prevent induction of LTM by cAMP analogue (Fig. 4). This finding is best explained if CaMKII interacts with AC by forming a multimolecular complex (see Discussion).

## Discussion

The roles of CaMKII in learning and memory have been extensively studied in rodents [1]. Once activated by Ca^2+^/CaM, CaMKII can maintain an active state by auto-phosphorylation after removal of Ca^2+^ and thus can act as a switch for memory retention in some systems of learning [2]. Knowledge of the roles of CaMKII in learning and memory in invertebrates, however, is limited. In this study, we showed that, in olfactory learning in crickets, injection of the CaMKII inhibitor KN-62 or KN-93 prior to conditioning impairs formation of 1-day memory (LTM), but it does not impair learning acquisition and formation of 1-hour memory (MTM), thus we suggest that CaMKII specifically participates in LTM formation in crickets. We should be cautious about possible side effects of CaMKII inhibitors: KN-62 might inhibit P2×7 receptor [36] and KN-93 might inhibit L-type calcium channel [37]. However, the common effect of KN-62 and KN-93 and their non-overlapping side effects strongly argue for their effect on CaMKII. KN-62 or KN93 inhibits Ca^2+^/CaM-dependent CaMKII but not Ca^2+^/CaM-insensitive, constitutively activated CaMKII [35], and thus the CaMKII involved in LTM formation should be a Ca^2+^/CaM-dependent type. We observed no impairment of LTM formation when KN62 was injected 20 min after conditioning, indicating that CaMKII activation is required at the time of conditioning. This observation, however, does not rule out the possibility that activation of CaMKII needs to be maintained for some time after conditioning for LTM formation, because KN-62 or KN-93 does not inhibit CaMKII when it is in an activated state [38].

The findings in crickets described above are in accordance with our recent findings in honey bees that KN-62 specifically impairs formation of protein synthesis-dependent LTM but not that of protein synthesis-independent MTM in olfactory conditioning [22]. Therefore, participation of CaMKII in the formation of protein synthesis-dependent LTM may be ubiquitous among different insect species. However, in the fruit-fly *Drosophila*, in which the molecular basis of learning and memory has been extensively studied [8], there has been no evidence suggesting participation of CaMKII in signaling cascades for activation of cAMP/PKA/CREB signaling system leading to protein synthesis-dependent LTM. More studies on the generality and specificity of the roles of CaMKII for LTM formation in different species of insects are needed.

For evaluation of the precise location of CaMKII in signaling cascades for LTM formation, we summarized some of the relevant findings in this study and in our previous studies [24], [25] in Table 2. As shown in Table 2, we have reported that an AC inhibitor (DDA) impairs induction of LTM by a Ca^2+^ ionophore (A23187), whereas a CaM inhibitor (W-7) does not impair LTM induction by an AC activator (forskolin), indicating that Ca^2+^/CaM activation is upstream of AC activation for LTM formation. In this study, we showed that KN-62 impairs LTM induction by a Ca^2+^ ionophore (ionomycin) but not that by a cAMP analogue.

**Table 2:** Summary of the effects of inhibitors on 30- or 60-min memory and 24-hr memory.

Memory investigated	Inhibitor (its target)		
	W-7 (CAM)	KN-62 (CaMKII)	DDA (AC)
30- or 60-min memory after multiple-trial conditioning	no effect	no effect	no effect
24-hr memory after multiple-trial conditioning	fully impaired	fully impaired	fully impaired
24-hr memory after calcium ionophore +1 trial conditioning	fully impaired	fully impaired	fully impaired
24-hr memory after forskolin +1 trial conditioning	no effect	fully impaired	fully impaired
24-hr memory after cAMP analogue +1 trial conditioning	no effect	no effect	no effect

CAM: calmodulin, AC: adenylyl cyclase, DDA: 2′5′-dideoxyadenosine.

Data for W7 and DDA experiments are from [24]; Data for KN-62 experiments are from this study.

**Figure 7:**
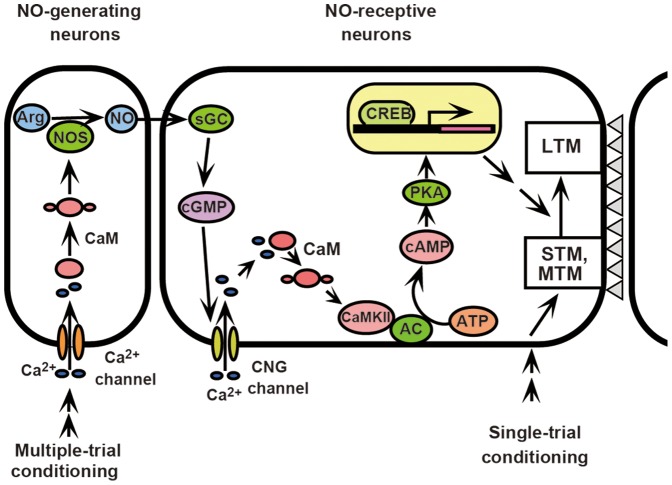
A model of the biochemical pathways for LTM formation in crickets. A model proposed for the biochemical pathways for olfactory LTM formation in crickets, in which our previous model [24], [25] was modified to account for findings in this study. Single-trial conditioning induces only short-term synaptic plasticity that underlies amnesic treatment-sensitive short-term memory (STM) and amnesic treatment-resistant mid-term memory (MTM) [24]. Multiple-trial conditioning activates NO/cGMP signaling, and this activates cyclic nucleotide-gated (CNG) channel, Ca^2+^/CaM, CaMKII and then adenylyl cyclase (AC)/cAMP/PKA signaling. This in turn activates cAMP responsive element-binding protein (CREB), which results in transcription and translation of genes that are necessary for achieving long-term plasticity of synaptic connection (a column of gray triangles) upon other neurons that underlies LTM. Thus, CaMKII intermediates between Ca^2+^ signaling and cAMP signaling.

On the basis of these findings, we propose that Ca^2+^/CaM activation leads to CaMKII activation and this in turn activates cAMP signaling for LTM formation. In Fig. 7, this proposal is incorporated into the signaling cascades for LTM formation we proposed in our previous studies [24], [25]. Our proposal of serial biochemical pathway is based on the assumption that inhibitors used in this study and in our previous studies affected the same neural networks, not in different networks acting in parallel for LTM formation. We assume this because we observed that all inhibitors used in this and in our previous studies [24], [25] induced complete impairment of LTM formation, with no significant level of 1-day memory retention: we expect partial inhibition of LTM formation if one of pathways acting in parallel is inhibited by the drugs. An alternative possibility is that CaM/CaMKII and AC/cAMP signaling systems work in parallel in different biochemical network and both play necessary roles for LTM formation. If this is the case, we expect that inhibition of either of the two signaling systems impairs LTM induction by activators of another system, but we observed that inhibition of CaMKII does not impair LTM induction by 8-br cAMP (Fig. 4). It should be cautioned, however, that there is a possibility that both of the two parallel systems need to be activated for LTM formation by 3-trial conditioning but not for LTM induction by activators, namely, synergistic effects of moderate activation of two parallel systems lead to LTM formation by 3-trial conditioning but excessive activation of AC/cAMP system leads to LTM formation without activation of CaM/CaMKII system in induction experiment. Future quantitative pharmacological analysis, as well as cellular and biochemical studies, are needed to better clarify the validity of our proposal.

Our observation that KN-62 or KN-93 impairs LTM induction by an AC activator (forskolin) is rather surprising, because KN-62 did not impair LTM induction by a cAMP analogue (see Table 2) and thus CaMKII should be upstream, not downstream, of the cAMP production in the pathway for LTM formation. These observations are best accounted for if CaMKII interacts with AC, probably by forming a macromolecular complex as it does with target molecules in mammals [1], [2], so that binding of KN-62 or KN-93 to CaMKII prevents activation of AC by forskolin, although immunohistochemical and biochemical studies are needed to confirm this speculation. Alternatively, CaMKII might inhibit phosphodiesterase (PDE), which degrades cAMP, so that inhibition of CaMKII activates PDE and prevents an increase in cAMP concentration even when AC is activated by forskolin. This possibility, however, is unlikely because it does not match our previous observation that a CaM inhibitor (W7), which should prevent activation of CaMKII, does not impair LTM induction by an AC activator (forskolin) (see Table 2). We thus propose that CaMKII stimulates cAMP production by activating AC for LTM formation in crickets (Fig. 7), a function previously not ascribed to CaMKII in any animals.

A notable feature of CaMKII is that it can maintain prolonged activation after it is activated by Ca^2+^/CaM. In honey bees, prolonged activation of PKA in the antennal lobe (primary olfactory center) for up to 90 sec is needed for LTM formation in olfactory conditioning [27]. Whether CaMKII acts as a molecular switch to maintain long-term activation of downstream cAMP signaling for LTM induction is a fascinating future subject.

We suggest that AC is a target molecule of CaMKII for LTM formation in crickets. Several biochemical types of AC isoforms have been identified in mice [39], fruit-flies [40] and honey bees [41]. So far, an AC type activated by CaMKII has not yet been reported in any animals, although a type inhibited by CaMKII has been reported in mice [42]. In crickets, AC plays critical roles in LTM formation but not in formation of short-term memory (STM) or MTM [24] as in honey bees [22]. In contrast, in the fruit-fly *Drosophila*, a type of AC (*rutabaga* AC) that is activated by Ca^2+^/CaM and G-protein plays critical roles in STM formation [8]. Characterization of biochemical type of AC that participates in LTM formation in crickets is awaited.

We suggest that CaMKII activates cAMP signaling for LTM formation in crickets, but it is evident that CaMKII plays diverse roles in insect learning. In courtship conditioning in fruit-flies, constitutively activated CaMKII in cholinergic neurons of the antennal lobe plays roles in behavioral change during training [16], [43], and CaMKII in neurons of the lateral protocerebrum or the central complex plays roles in acquisition of memory [15]. In olfactory learning in fruit-flies, it has been reported that CREB-dependent transcription and translation of CaMKII proteins in Kenyon cells of the mushroom body [19], [20] or dorsal-anterior-lateral (DAL) neurons [44] are necessary for LTM formation. In cockroaches, the density of the activated (phosphorylated) form of CaMKII is increased by conditioning of odor with a visual target in the calyx of the mushroom body, where Kenyon cells receive synaptic inputs [21]. It is most likely that CaMKII is a critical molecule in many systems of learning in insects, as is in mammals [1], [2].

One of the important subjects that we should address in the future is to clarify brain areas and types of neurons in which activation of CaMKII leads to LTM formation. In fruit-flies, the participation of Kenyon cells of the mushroom body in LTM formation is well established [8], and this most likely applies to crickets, because we observed that mRNA of NO synthase (NOS) and soluble guanylyl cyclase (sGC), key molecules for LTM formation in crickets, are densely distributed in outer and inner Kenyon cells, respectively [45]. It has also been reported that enzymatic activity of CaMKII is enriched in Kenyon cells in fruit-flies [18], [46], honey bees [47], [48] and cockroaches [21]. Therefore, establishment of quantification technique of the enzymatic activity of CaMKII in the cricket brain may lead to better elucidation of brain mechanisms of LTM formation.

## References

[1] Coultrap SJ, Bayer KU (2012) CaMKII regulation in information processing and storage. Trends Neurosci 35: 607–618. 22717267 10.1016/j.tins.2012.05.003 PMC3461103

[2] Lisman J, Yasuda R, Raghavachari S (2012) Mechanisms of CaMKII action in long-term potentiation. Nat Rev Neurosci 13: 169–182. 22334212 10.1038/nrn3192 PMC4050655

[3] Lisman J, Schulman H, Cline H (2002) The molecular basis of CaMKII function in synaptic and behavioural memory. Nat Rev Neurosci 3: 175–190. 11994750 10.1038/nrn753

[4] Silva AJ, Paylor R, Wehner JM, Tonegawa S (1992a) Deficient hippocampal long-term potentiation in α-calcium-calmodulin Kinase II mutant mice. Science 257: 201–206. 1378648 10.1126/science.1378648

[5] Silva AJ, Stevens CF, Tonegawa S, Wang Y (1992b) Impaired spatial learning in α-calcium-calmodulin Kinase II mutant mice. Science 257: 206–211. 1321493 10.1126/science.1321493

[6] Giese KP, Fedorov NB, Filipkowski RK, Silva AJ (1998) Autophosphorylation at Thr286 of the alpha calcium-calmodulin kinase II in LTP and learning. Science 279: 870–873. 9452388 10.1126/science.279.5352.870

[7] Kandel ER (2001) The molecular biology of memory storage: a dialogue between genes and synapses. Science 294: 1030–1038. 11691980 10.1126/science.1067020

[8] Davis RL (2011) Traces of *Drosophila* memory. Neuron 70: 8–19. 21482352 10.1016/j.neuron.2011.03.012 PMC3374581

[9] Giurfa M, Sandoz JC (2012) Invertebrate learning and memory: fifty years of olfactory conditioning of the proboscis extension response in honeybees. Learn Mem 19: 54–66. 22251890 10.1101/lm.024711.111

[10] Mizunami M, Unoki S, Mori Y, Hirashima D, Hatano A, et al (2009) Roles of octopaminergic and dopaminergic neurons in appetitive and aversive memory recall in an insect. BMC Biology 7: 46. 19653886 10.1186/1741-7007-7-46 PMC2729297

[11] Mizunami M, Matsumoto Y, Watanabe H, Nishino H (2013) Chapter 41: Olfactory and visual learning in cockroaches and crickets. In: Invertebrate Learning and Memory, eds by R Menzel and PR Benjamin, Springer, pp.547–558.

[12] Luk CC, Naruo H, Prince D, Hassan A, Doran SA, et al (2011) A novel form of presynaptic CaMKII-dependent short-term potentiation between *Lymnaea* neurons. Eur J Neurosci 34: 569–577. 21749498 10.1111/j.1460-9568.2011.07784.x

[13] Antonov I, Kandel ER, Hawkins RD (2010) Presynaptic and postsynaptic mechanisms of synaptic plasticity and metaplasticity during intermediate-term memory formation in *Aplysia* . J Neurosci 30: 5781–5791. 20410130 10.1523/JNEUROSCI.4947-09.2010 PMC6632334

[14] Wan H, Mackay B, Iqbal H, Naskar S, Kemenes G (2010) Delayed intrinsic activation of an NMDA-independent CaM-kinase II in a critical time window is necessary for late consolidation of an associative memory. J Neurosci 30: 56–63. 20053887 10.1523/JNEUROSCI.2577-09.2010 PMC6632524

[15] Joiner MA, Griffith LC (1999) Mapping of the anatomical circuit of CaM kinase-dependent courtship conditioning in *Drosophila* . Learn Mem 6: 177–192. 10327242 PMC311288

[16] Mehren JE, Griffith LC (2004) Calcium-independent calcium/calmodulin-dependent protein kinase II in the adult *Drosophila* CNS enhances the training of pheromonal cues. J Neurosci 24: 10584–10593. 15564574 10.1523/JNEUROSCI.3560-04.2004 PMC6730130

[17] Heisenberg M (2003) Mushroom body memoir: from maps to models. Nature Reviews Neuroscience 4: 266–275. 12671643 10.1038/nrn1074

[18] Ashraf SI, McLoon AL, Sclarsic SM, Kunes S (2006) Synaptic protein synthesis associated with memory is regulated by the RISC pathway in *Drosophila* . Cell 124: 191–205. 16413491 10.1016/j.cell.2005.12.017

[19] Akalal DB, Yu D, Davis RL (2010) A late-phase, long-term memory trace forms in the γ neurons of *Drosophila* mushroom bodies after olfactory classical conditioning. J Neurosci 30: 16699–16708. 21148009 10.1523/JNEUROSCI.1882-10.2010 PMC3380342

[20] Malik BR, Gillespie JM, Hodge JJ (2013) CASK and CaMKII function in the mushroom body α'/β' neurons during *Drosophila* memory formation. Front Neural Circuits 7: 52. 23543616 10.3389/fncir.2013.00052 PMC3608901

[21] Lent DD, Pintér M, Strausfeld NJ (2007) Learning with half a brain. Dev Neurobiol 67: 740–751. 17443821 10.1002/dneu.20374

[22] Matsumoto Y, Sandoz JC, Devaud JM, Lormant F, Mizunami M, et al (2014) Cyclic nucleotide–gated channels, Calmodulin, adenylyl cyclase and calcium/calmodulin-dependent protein kinase II are required for late but not early long-term memory formation in the honey bee. Learn Mem 21: 272–286. 24741108 10.1101/lm.032037.113 PMC3994501

[23] Yin JCP, Del Vecchio M, Zhou H, Tully T (1995) CREB as a memory modulator: induced expression of a dCREB2 activator isoform enhances long-term memory in *Drosophila* . Cell 81: 107–115. 7720066 10.1016/0092-8674(95)90375-5

[24] Matsumoto Y, Unoki S, Aonuma H, Mizunami M (2006) Critical role of nitric oxide-cGMP cascade in the formation of cAMP-dependent long-term memory. Learn Mem 13: 35–44. 16452652 10.1101/lm.130506 PMC1360131

[25] Matsumoto Y, Hatano A, Unoki S, Mizunami M (2009) Stimulation of the cAMP system by the nitric oxide-cGMP system underlying the formation of long-term memory in an insect. Neurosci Lett 467: 81–85. 19818830 10.1016/j.neulet.2009.10.008

[26] Müller U (1996) Inhibition of nitric oxide synthase impairs a distinct form of long-term memory in the honeybee, *Apis mellifera* . Neuron 16: 541–549. 8785051 10.1016/s0896-6273(00)80073-2

[27] Müller U (2000) Prolonged activation of cAMP-dependent protein kinase during conditioning induces long-term memory in honeybees. Neuron 27: 159–168. 10939339 10.1016/s0896-6273(00)00017-9

[28] Matsumoto CS, Kuramochi T, Matsumoto Y, Watanabe H, Nishino H, et al (2013a) Participation of NO signaling in formation of long-term memory in salivary conditioning of the cockroach. Neurosci Lett 541: 4–8. 23333539 10.1016/j.neulet.2013.01.010

[29] Matsumoto Y, Hirashima D, Terao K, Mizunami M (2013b) Roles of NO signaling in long-term memory formation in visual learning in an insect. PLoS One 8: e68538. 23894314 10.1371/journal.pone.0068538 PMC3722230

[30] Matsumoto Y, Mizunami M (2002) Temporal determinants of long-term retention of olfactory memory in the cricket *Gryllus bimaculatus* . J Exp Biol 205: 1429–1437. 11976354 10.1242/jeb.205.10.1429

[31] Unoki S, Matsumoto Y, Mizunami M (2005) Participation of octopaminergic reward system and dopaminergic punishment system in insect olfactory learning revealed by pharmacological study. Eur J Neurosci 22: 1409–1416. 16190895 10.1111/j.1460-9568.2005.04318.x

[32] Matsumoto Y, Noji S, Mizunami M (2003) Time course of protein synthesis-dependent phase of olfactory memory in the cricket *Gryllus bimaculatus* . Zool Sci 20: 409–416. 12719642 10.2108/zsj.20.409

[33] Shanavas A, Dutta-Gupta A, Murthy CRK (1998) Identification, characterization, immunocytochemical localization, and developmental changes in the activity of calcium/calmodulin-dependent protein kinase II in the CNS of *Bombyx mori* during postembryonic development. J Neurochem 70: 1644–1651. 9523582 10.1046/j.1471-4159.1998.70041644.x

[34] Burkert P, Duch C (2006) Developmental changes of CaMKII localization, activity and function during postembryonic CNS remodelling in *Manduca sexta* . Eur J Neurosci 23: 335–349. 16420442 10.1111/j.1460-9568.2005.04562.x

[35] Chavez-Noriega LE, Stevens CF (1992) Modulation of synaptic efficacy in field CA1 of the rat hippocampus by forskolin. Brain Res 574: 85–92. 1379110 10.1016/0006-8993(92)90803-h

[36] Gunosewoyo H, Coster MJ, Kassiou M (2007) Molecular probes for P2×7 receptor studies. Curr Med Chem 14: 1505–1523. 17584060 10.2174/092986707780831023

[37] Gao L, Blair LA, Marshall J (2006) CaMKII-independent effects of KN93 and its inactive analog KN92: reversible inhibition of L-type calcium channels. Biochem Biophys Res Commun 345: 1606–1610. 16730662 10.1016/j.bbrc.2006.05.066

[38] Tokumitsu H, Chijiwa T, Hagiwara M, Mitzutani A, Terasawa M, et al (1990) KN-62, 1-[N, O-Bis (5-isoquinolinesulfonyl)-N-methyl-L-tyrosyl]-4-phenylpiperazine, a specific inhibitor of Ca^2+^/calmodulin-dependent protein kinase II. J Biol Chem 265: 4315–4320. 2155222

[39] Sunahara RK, Taussig R (2002) Isoforms of mammalian adenylyl cyclase: Multiplicities of signaling. Mol Interv 2: 168–184. 14993377 10.1124/mi.2.3.168

[40] Cann MJ, Levin LR (2002) Identification of transmembrane adenylyl cyclase isoforms. Methods Enzymol 345: 150–159. 11665602 10.1016/s0076-6879(02)45014-8

[41] Balfanz S, Ehling P, Wachten S, Jordan N, Erber J, et al (2012) Functional characterization of transmembrane adenylyl cyclases from the honeybee brain. Insect Biochem Mol Biol 42: 435–445. 22426196 10.1016/j.ibmb.2012.02.005

[42] Wei J, Wayman G, Storm DR (1996) Phosphorylation and inhibition of type III adenylyl cyclase by calmodulin-dependent protein kinase II in vivo. J Biochem 271: 24231–24235. 10.1074/jbc.271.39.24231 8798667

[43] Mehren JE, Griffith LC (2006) Cholinergic neurons mediate CaMK II-dependent enhancement of courtship suppression. Learn Mem 13: 686–689. 17101876 10.1101/lm.317806

[44] Chen CC, Wu JK, Lin HW, Pai TP, Fu TF, et al (2012) Visualizing long-term memory formation in two neurons of the *Drosophila* brain. Science 335: 678–685. 22323813 10.1126/science.1212735

[45] Takahashi T, Hamada A, Miyawaki K, Matsumoto Y, Mito T, et al (2009) Systemic RNA interference for the study of learning and memory in an insect. J Neurosci Methods 179: 9–15. 19437615 10.1016/j.jneumeth.2009.01.002

[46] Takamatsu Y, Kishimoto Y, Ohsako S (2003) Immunohistochemical study of Ca2+/calmodulin-dependent kinase II in the *Drosophila* brain using a specific monoclonal antibody. Brain Res 974: 99–106. 12742628 10.1016/s0006-8993(03)02562-9

[47] Kamikouchi A, Takeuchi H, Sawata M, Natori S, Kubo T (2000) Concentrated expression of Ca2+/calmodulin-dependent kinase II and protein kinase C in the mushroom bodies of the honeybee *Apis mellifera* L. J Comp Neurol 417: 501–510. 10701869 10.1002/(sici)1096-9861(20000221)417:4<501::aid-cne8>3.0.co;2-4

[48] Pasch E, Muenz TS, Rössler W (2011) CaMKII is differentially localized in synaptic regions of Kenyon cells within the mushroom bodies of the honeybee brain. J Comp Neurol 519: 3700–3712. 21674485 10.1002/cne.22683

